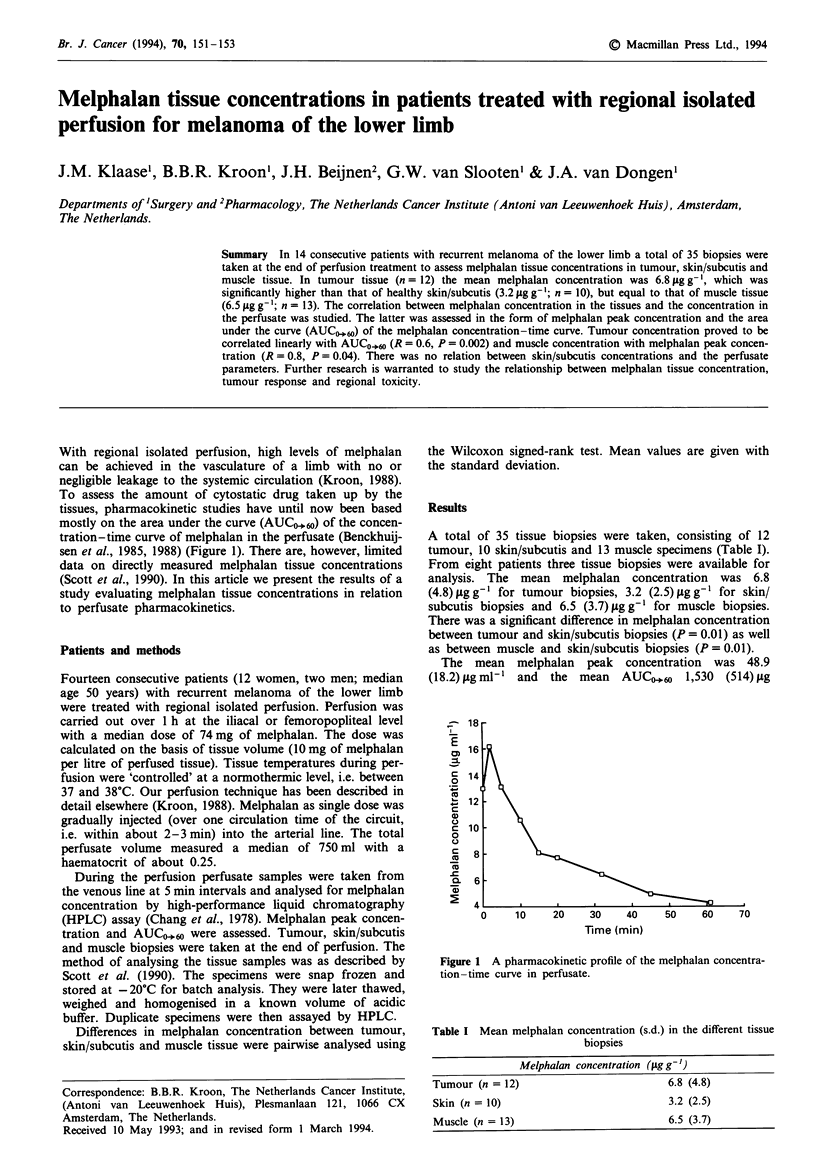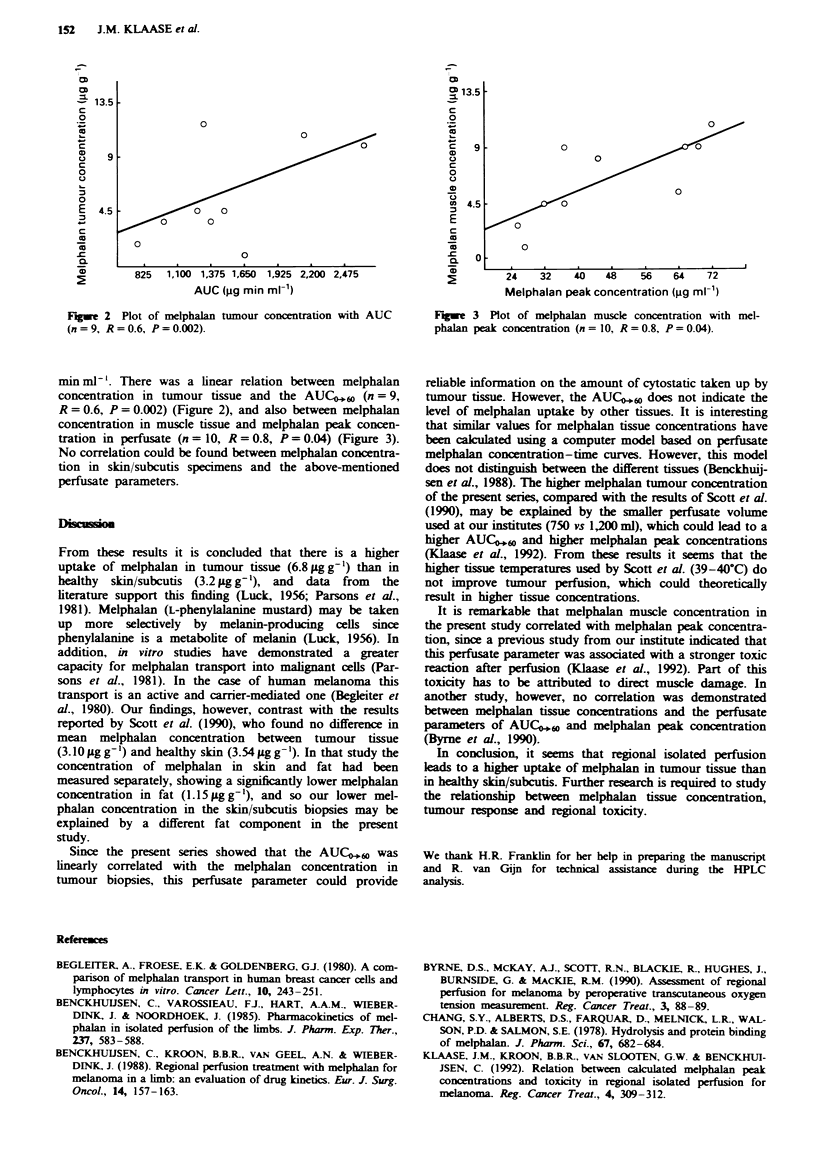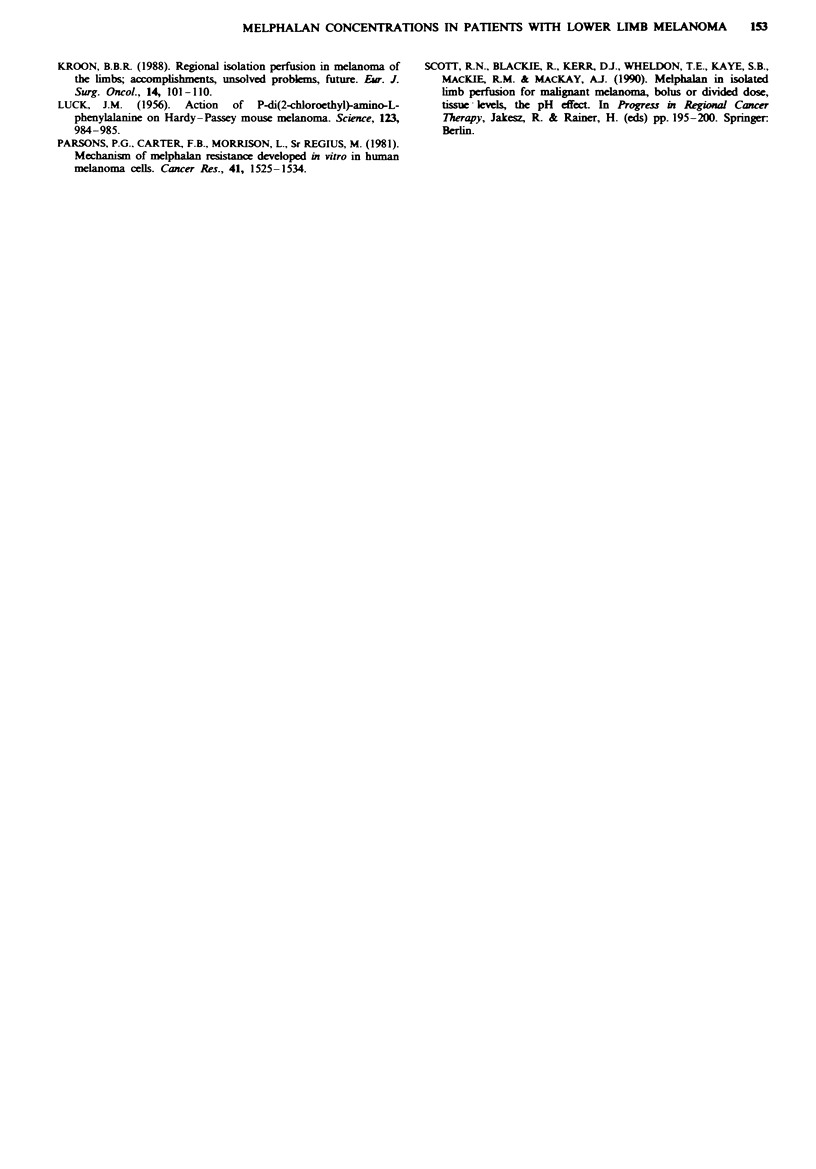# Melphalan tissue concentrations in patients treated with regional isolated perfusion for melanoma of the lower limb.

**DOI:** 10.1038/bjc.1994.266

**Published:** 1994-07

**Authors:** J. M. Klaase, B. B. Kroon, J. H. Beijnen, G. W. van Slooten, J. A. van Dongen

**Affiliations:** Department of Surgery, The Netherlands Cancer Institute (Antoni van Leeuwenhoek Huis), Amsterdam.

## Abstract

In 14 consecutive patients with recurrent melanoma of the lower limb a total of 35 biopsies were taken at the end of perfusion treatment to assess melphalan tissue concentrations in tumour, skin/subcutis and muscle tissue. In tumour tissue (n = 12) the mean melphalan concentration was 6.8 micrograms g-1, which was significantly higher than that of healthy skin/subcutis (3.2 micrograms g-1; n = 10), but equal to that of muscle tissue (6.5 micrograms g-1; n = 13). The correlation between melphalan concentration in the tissues and the concentration in the perfusate was studied. The latter was assessed in the form of melphalan peak concentration and the area under the curve (AUC0-->60) of the melphalan concentration-time curve. Tumour concentration proved to be correlated linearly with AUC0-->60 (R = 0.6, P = 0.002) and muscle concentration with melphalan peak concentration (R = 0.8, P = 0.04). There was no relation between skin/subcutis concentrations and the perfusate parameters. Further research is warranted to study the relationship between melphalan tissue concentration, tumour response and regional toxicity.


Br. J. Cancer (1994), 70, 151–153 © Macmillan Press Ltd., 1994

### Melphalan tissue concentrations in patients treated with regional isolated perfusion for melanoma of the lower limb

J.M. Klaase[1], B.B.R. Kroon[1], J.H. Beijnen[2], G.W. van Slooten[1] & J.A. van Dongen[1]

*Departments of [1]Surgery and [2]Pharmacology, The Netherlands Cancer Institute (Antoni van Leeuwenhoek Huis), Amsterdam, The Netherlands.*

**Summary** In 14 consecutive patients with recurrent melanoma of the lower limb a total of 35 biopsies were taken at the end of perfusion treatment to assess melphalan tissue concentrations in tumour, skin/subcutis and muscle tissue. In tumour tissue ($n = 12$) the mean melphalan concentration was 6.8 $\mu g\ g^{-1}$, which was significantly higher than that of healthy skin/subcutis (3.2 $\mu g\ g^{-1}$; $n = 10$), but equal to that of muscle tissue (6.5 $\mu g\ g^{-1}$; $n = 13$). The correlation between melphalan concentration in the tissues and the concentration in the perfusate was studied. The latter was assessed in the form of melphalan peak concentration and the area under the curve ($AUC_{0\rightarrow60}$) of the melphalan concentration–time curve. Tumour concentration proved to be correlated linearly with $AUC_{0\rightarrow60}$ ($R = 0.6$, $P = 0.002$) and muscle concentration with melphalan peak concentration ($R = 0.8$, $P = 0.04$). There was no relation between skin/subcutis concentrations and the perfusate parameters. Further research is warranted to study the relationship between melphalan tissue concentration, tumour response and regional toxicity.

With regional isolated perfusion, high levels of melphalan can be achieved in the vasculature of a limb with no or negligible leakage to the systemic circulation (Kroon, 1988). To assess the amount of cytostatic drug taken up by the tissues, pharmacokinetic studies have until now been based mostly on the area under the curve ($AUC_{0\rightarrow60}$) of the concentration–time curve of melphalan in the perfusate (Benckhuijsen *et al.*, 1985, 1988) (Figure 1). There are, however, limited data on directly measured melphalan tissue concentrations (Scott *et al.*, 1990). In this article we present the results of a study evaluating melphalan tissue concentrations in relation to perfusate pharmacokinetics.

### Patients and methods

Fourteen consecutive patients (12 women, two men; median age 50 years) with recurrent melanoma of the lower limb were treated with regional isolated perfusion. Perfusion was carried out over 1 h at the iliacal or femoropopliteal level with a median dose of 74 mg of melphalan. The dose was calculated on the basis of tissue volume (10 mg of melphalan per litre of perfused tissue). Tissue temperatures during perfusion were 'controlled' at a normothermic level, i.e. between 37 and 38°C. Our perfusion technique has been described in detail elsewhere (Kroon, 1988). Melphalan as single dose was gradually injected (over one circulation time of the circuit, i.e. within about 2–3 min) into the arterial line. The total perfusate volume measured a median of 750 ml with a haematocrit of about 0.25.

During the perfusion perfusate samples were taken from the venous line at 5 min intervals and analysed for melphalan concentration by high-performance liquid chromatography (HPLC) assay (Chang *et al.*, 1978). Melphalan peak concentration and $AUC_{0\rightarrow60}$ were assessed. Tumour, skin/subcutis and muscle biopsies were taken at the end of perfusion. The method of analysing the tissue samples was as described by Scott *et al.* (1990). The specimens were snap frozen and stored at −20°C for batch analysis. They were later thawed, weighed and homogenised in a known volume of acidic buffer. Duplicate specimens were then assayed by HPLC.

Differences in melphalan concentration between tumour, skin/subcutis and muscle tissue were pairwise analysed using the Wilcoxon signed-rank test. Mean values are given with the standard deviation.

### Results

A total of 35 tissue biopsies were taken, consisting of 12 tumour, 10 skin/subcutis and 13 muscle specimens (Table I). From eight patients three tissue biopsies were available for analysis. The mean melphalan concentration was 6.8 (4.8) $\mu g\ g^{-1}$ for tumour biopsies, 3.2 (2.5) $\mu g\ g^{-1}$ for skin/subcutis biopsies and 6.5 (3.7) $\mu g\ g^{-1}$ for muscle biopsies. There was a significant difference in melphalan concentration between tumour and skin/subcutis biopsies ($P = 0.01$) as well as between muscle and skin/subcutis biopsies ($P = 0.01$).

The mean melphalan peak concentration was 48.9 (18.2) $\mu g\ ml^{-1}$ and the mean $AUC_{0\rightarrow60}$ 1,530 (514) $\mu g$

**Figure 1** A pharmacokinetic profile of the melphalan concentration–time curve in perfusate.

**Table I** Mean melphalan concentration (s.d.) in the different tissue biopsies

| | *Melphalan concentration* ($\mu g\ g^{-1}$) |
|---|---|
| Tumour ($n = 12$) | 6.8 (4.8) |
| Skin ($n = 10$) | 3.2 (2.5) |
| Muscle ($n = 13$) | 6.5 (3.7) |

Correspondence: B.B.R. Kroon, The Netherlands Cancer Institute, (Antoni van Leeuwenhoek Huis), Plesmanlaan 121, 1066 CX Amsterdam, The Netherlands.
Received 10 May 1993; and in revised form 1 March 1994.

**Figure 2** Plot of melphalan tumour concentration with AUC ($n = 9$, $R = 0.6$, $P = 0.002$).

**Figure 3** Plot of melphalan muscle concentration with melphalan peak concentration ($n = 10$, $R = 0.8$, $P = 0.04$).

min $ml^{-1}$. There was a linear relation between melphalan concentration in tumour tissue and the $AUC_{0\rightarrow 60}$ ($n = 9$, $R = 0.6$, $P = 0.002$) (Figure 2), and also between melphalan concentration in muscle tissue and melphalan peak concentration in perfusate ($n = 10$, $R = 0.8$, $P = 0.04$) (Figure 3). No correlation could be found between melphalan concentration in skin/subcutis specimens and the above-mentioned perfusate parameters.

### Discussion

From these results it is concluded that there is a higher uptake of melphalan in tumour tissue (6.8 $\mu g\ g^{-1}$) than in healthy skin/subcutis (3.2 $\mu g\ g^{-1}$), and data from the literature support this finding (Luck, 1956; Parsons *et al.*, 1981). Melphalan (L-phenylalanine mustard) may be taken up more selectively by melanin-producing cells since phenylalanine is a metabolite of melanin (Luck, 1956). In addition, *in vitro* studies have demonstrated a greater capacity for melphalan transport into malignant cells (Parsons *et al.*, 1981). In the case of human melanoma this transport is an active and carrier-mediated one (Begleiter *et al.*, 1980). Our findings, however, contrast with the results reported by Scott *et al.* (1990), who found no difference in mean melphalan concentration between tumour tissue (3.10 $\mu g\ g^{-1}$) and healthy skin (3.54 $\mu g\ g^{-1}$). In that study the concentration of melphalan in skin and fat had been measured separately, showing a significantly lower melphalan concentration in fat (1.15 $\mu g\ g^{-1}$), and so our lower melphalan concentration in the skin/subcutis biopsies may be explained by a different fat component in the present study.

Since the present series showed that the $AUC_{0\rightarrow 60}$ was linearly correlated with the melphalan concentration in tumour biopsies, this perfusate parameter could provide reliable information on the amount of cytostatic taken up by tumour tissue. However, the $AUC_{0\rightarrow 60}$ does not indicate the level of melphalan uptake by other tissues. It is interesting that similar values for melphalan tissue concentrations have been calculated using a computer model based on perfusate melphalan concentration–time curves. However, this model does not distinguish between the different tissues (Benckhuijsen *et al.*, 1988). The higher melphalan tumour concentration of the present series, compared with the results of Scott *et al.* (1990), may be explained by the smaller perfusate volume used at our institutes (750 *vs* 1,200 ml), which could lead to a higher $AUC_{0\rightarrow 60}$ and higher melphalan peak concentrations (Klaase *et al.*, 1992). From these results it seems that the higher tissue temperatures used by Scott *et al.* (39–40°C) do not improve tumour perfusion, which could theoretically result in higher tissue concentrations.

It is remarkable that melphalan muscle concentration in the present study correlated with melphalan peak concentration, since a previous study from our institute indicated that this perfusate parameter was associated with a stronger toxic reaction after perfusion (Klaase *et al.*, 1992). Part of this toxicity has to be attributed to direct muscle damage. In another study, however, no correlation was demonstrated between melphalan tissue concentrations and the perfusate parameters of $AUC_{0\rightarrow 60}$ and melphalan peak concentration (Byrne *et al.*, 1990).

In conclusion, it seems that regional isolated perfusion leads to a higher uptake of melphalan in tumour tissue than in healthy skin/subcutis. Further research is required to study the relationship between melphalan tissue concentration, tumour response and regional toxicity.

We thank H.R. Franklin for her help in preparing the manuscript and R. van Gijn for technical assistance during the HPLC analysis.

### References

BEGLEITER, A., FROESE, E.K. & GOLDENBERG, G.J. (1980). A comparison of melphalan transport in human breast cancer cells and lymphocytes *in vitro*. *Cancer Lett.*, **10**, 243–251.

BENCKHUIJSEN, C., VAROSSIEAU, F.J., HART, A.A.M., WIEBERDINK, J. & NOORDHOEK, J. (1985). Pharmacokinetics of melphalan in isolated perfusion of the limbs. *J. Pharm. Exp. Ther.*, **237**, 583–588.

BENCKHUIJSEN, C., KROON, B.B.R., VAN GEEL, A.N. & WIEBERDINK, J. (1988). Regional perfusion treatment with melphalan for melanoma in a limb: an evaluation of drug kinetics. *Eur. J. Surg. Oncol.*, **14**, 157–163.

BYRNE, D.S., MCKAY, A.J., SCOTT, R.N., BLACKIE, R., HUGHES, J., BURNSIDE, G. & MACKIE, R.M. (1990). Assessment of regional perfusion for melanoma by peroperative transcutaneous oxygen tension measurement. *Reg. Cancer Treat.*, **3**, 88–89.

CHANG, S.Y., ALBERTS, D.S., FARQUAR, D., MELNICK, L.R., WALSON, P.D. & SALMON, S.E. (1978). Hydrolysis and protein binding of melphalan. *J. Pharm. Sci.*, **67**, 682–684.

KLAASE, J.M., KROON, B.B.R., VAN SLOOTEN, G.W. & BENCKHUIJSEN, C. (1992). Relation between calculated melphalan peak concentrations and toxicity in regional isolated perfusion for melanoma. *Reg. Cancer Treat.*, **4**, 309–312.

KROON, B.B.R. (1988). Regional isolation perfusion in melanoma of the limbs; accomplishments, unsolved problems, future. *Eur. J. Surg. Oncol.*, **14**, 101-110.

LUCK, J.M. (1956). Action of P-di(2-chloroethyl)-amino-L-phenylalanine on Hardy-Passey mouse melanoma. *Science*, **123**, 984-985.

PARSONS, P.G., CARTER, F.B., MORRISON, L., Sr REGIUS, M. (1981). Mechanism of melphalan resistance developed *in vitro* in human melanoma cells. *Cancer Res.*, **41**, 1525-1534.

SCOTT, R.N., BLACKIE, R., KERR, D.J., WHELDON, T.E., KAYE, S.B., MACKIE, R.M. & MACKAY, A.J. (1990). Melphalan in isolated limb perfusion for malignant melanoma, bolus or divided dose, tissue levels, the pH effect. In *Progress in Regional Cancer Therapy*, Jakesz, R. & Rainer, H. (eds) pp. 195-200. Springer: Berlin.